# Biomass Seedling Trays Drive Rhizosphere Microbiome Restructuring and PGPR Enrichment in Tomato

**DOI:** 10.3390/plants15101486

**Published:** 2026-05-13

**Authors:** Jiayun Zhang, Xiangyu Zhang, Qiang Chen

**Affiliations:** College of Resources, Sichuan Agricultural University, Chengdu 611130, China; zhangjiayun2026@126.com (J.Z.); zxy18180765055@126.com (X.Z.)

**Keywords:** tomato, rhizosphere microbial community structure, PGPR, biomass seedling tray

## Abstract

Tomato (*Solanum lycopersicum*) is a globally important high-value cash crop. However, long-term continuous cropping causes frequent soil-borne diseases and soil microecological imbalance, while overreliance on chemical pesticides leads to pesticide residues and water eutrophication. Plant growth-promoting rhizobacteria (PGPR) are key resources for addressing tomato cultivation challenges, with their functions partly depending on the rhizosphere microenvironment inherently shaped by seedling tray materials. Using rhizosphere soil and substrates of tomato at different growth stages under biomass (BM) and plastic (PM) seedling tray treatments, this study combined culture-independent and culture-dependent techniques to analyze microbial community characteristics and screen high-efficiency PGPR. Results showed that pH and available nitrogen drove microbial community assembly. BM significantly enriched beneficial taxa (e.g., *Trichoderma* and *Bacillus*) and enhanced culturable microbial abundance and genetic diversity, while PM enriched potential pathogens (e.g., *Fusarium* and *Pyrenochaeta*). The multifunctional strain S25095 from BM, with phosphate-solubilizing, potassium-solubilizing, and indole-3-acetic acid (IAA)-producing abilities, significantly promoted tomato shoot and root growth, outperforming single-functional strains and synthetic consortia. This study reveals the effects of growth stages and seedling tray treatments on tomato rhizosphere microorganisms, providing valuable PGPR resources for tomato cultivation.

## 1. Introduction

*Solanum lycopersicum*, also known as the tomato, is an annual herbaceous plant belonging to the genus *Solanum* in the family Solanaceae. It originated from the region spanning the Andes Mountains in South America to Mexico, was introduced to Europe in the 16th century, and has since been gradually cultivated worldwide [1]. Tomato fruits are highly nutritious, rich in lycopene [2], sugars, organic acids, various vitamins, and mineral elements [3]. According to the latest statistical data from the Food and Agriculture Organization (FAO) [4], the global tomato planting area reaches 5.12 million hectares, with a yield of 36.8 tons per hectare.

However, under intensive cultivation patterns, long-term continuous cropping disrupts soil ecosystems and triggers frequent outbreaks of soil-borne diseases (e.g., bacterial wilt, fusarium wilt), which severely affect tomato yield and quality, hindering the high-quality development of the industry [5]. To address these issues, existing control measures mainly include agricultural, physical, and chemical approaches [6]. For instance, a 4-year field experiment in Italy showed that a reasonable crop rotation system can improve soil microbial community structure and reduce tomato diseases [7]. Hilariòn Vàsquez et al. found that treating tomatoes with UV-C radiation at 0.85 kJ/m^2^ can increase phenylalanine ammonia-lyase (PAL) activity and bound phenolic content, significantly enhancing tomato leaves’ resistance to gray mold [8]. In addition, a greenhouse experiment in Fangshan District, Beijing, demonstrated that soil fumigation with 1,3-dichloropropene inhibited *Fusarium* and *Phytophthora* by over 75%, increasing tomato yield by 29.1% in the early harvest stage [9]. All of these measures can improve tomato yield and disease resistance to a certain extent. Nevertheless, each of these traditional control methods has its own limitations and challenges. Although crop rotation can improve soil microbial communities, its large-scale promotion is often restricted by land resources and the rigid constraints of cash crop planting plans under highly intensive and specialized production models [10]. Physical methods such as UV-C radiation are environmentally friendly, but their control effects are significantly influenced by equipment costs, operational standards, and environmental conditions [11,12]. Chemical measures act quickly and effectively inhibit pathogens, but long-term use tends to simplify soil microorganisms, increase pathogen resistance, and pose risks such as pesticide residues, environmental pollution, and threats to operators’ health [13,14,15]. This is contrary to the concept of sustainable agricultural development.

In the context of global climate change, the increasing frequency of extreme weather events and soil degradation poses significant challenges to agricultural sustainability [16]. Developing green, low-carbon, and resilient seedling technologies is therefore critical for climate adaptation and food security. The seedling stage is critical for intensive tomato production, and substrate and container materials directly shape the rhizosphere microenvironment and microbial community structure [17,18]. Commonly used plastic trays show poor permeability and degradability, hindering aeration, water transport, and colonization by beneficial microorganisms [19,20]. Biomass-based trays, with good porosity, degradability, and slow-release capacity, can optimize the rhizosphere microecology and enrich beneficial microbes [21]. This has become a key direction of green seedling technologies. A systematic analysis of the effects of different trays on rhizosphere microbial communities helps to clarify the establishment of rhizosphere microecology and achieve healthy tomato production.

Different seedling methods form distinct rhizosphere microenvironments, affecting the composition and growth-promoting potential of functional microorganisms. With a growing emphasis on green agriculture and food safety, exploring and utilizing efficient PGPR is crucial for green control [22]. PGPR functions via multiple mechanisms, including niche competition [23], secretion of antimicrobial substances [24], induction of systemic resistance [25], and growth promotion [26]. Through these mechanisms, PGPR enable green control of soil-borne diseases. Additionally, PGPR improve rhizosphere nutrient cycling, enhance soil enzyme activities [27], promote beneficial microbial colonization [28], stimulate root development, boost nutrient uptake, and regulate phytohormone balance [29]. These effects collectively enhance soil microecological stability, increase tomato yield, and improve fruit nutritional quality. The application of PGPR provides an effective path for the high-quality development of the tomato industry with coordinated ecological and economic benefits [30].

In the era of agricultural green transition, exploiting beneficial rhizosphere microorganisms is vital for sustainable agriculture [31]. We hypothesized that, compared with plastic seedling trays, biomass seedling trays restructure the rhizosphere microbial community by improving the rhizosphere microenvironment, enriching beneficial bacteria such as PGPR, and ultimately promoting tomato growth. To test this hypothesis, this study used rhizosphere soil and nursery substrates of tomato at different growth stages and with different seedling trays as materials. High-throughput sequencing and culture-dependent methods were applied to systematically analyze the structure and genetic diversity of both culturable and unculturable rhizosphere microbial communities. Culturable bacteria were then screened for phosphate-solubilizing, potassium-solubilizing, and indole-3-acetic acid (IAA)-producing growth-promoting functions, and differences in growth-promoting abilities among strains from different seedling trays were compared. Finally, efficient strains were selected for pot experiments to verify their growth-promoting effects on tomato, providing a theoretical basis and microbial resources for green tomato cultivation and soil ecological health maintenance.

## 2. Materials and Methods

### 2.1. Test Materials and Collection

Samples were collected at a tomato production base in Wanqiu Yi Township, Miyi County, Panzhihua City, Sichuan Province (27°05′ N, 102°09′ E, altitude 1149 m). Two treatments were established: biomass (BM) seedling tray and plastic (PM) seedling tray. Both treatments were placed in the same experimental field with a consistent initial soil background to eliminate interference from soil heterogeneity. The biomass trays used in this study were made from corn stalk powder (45%) and corn cob powder (55%) inoculated with *Ganoderma lucidum* for fermentation; had an organic matter content of 642 g/kg; and contained certain amounts of nitrogen, phosphorus, and potassium [32] and were compared with plastic trays. Tomato seedlings were grown in trays for one month and then transplanted to the field, where both BM and PM treatments received identical management. Each treatment was set up with 30 replicates. Sampling was conducted at three growth stages: the seedling stage A (15 September 2024), the early fruiting stage B (27 October 2024), and the peak fruiting stage C (6 March 2025). At each time point, four uniformly growing plants were randomly selected from each treatment, with each plant serving as an independent biological replicate. A total of eight types of samples were collected (Table 1). Shoots were removed, and the roots with attached soil were placed into sterile bags, labeled, and transported to the laboratory in an ice box. After gently shaking off loose non-rhizosphere soil, the rhizosphere soil within 2 mm of the root surface and the seedling substrate were collected using a sterile brush [33]. For each biological replicate, one aliquot was stored at −80 °C for uncultured microbial analysis, and the other was stored at 4 °C for isolation of culturable microorganisms.

### 2.2. Determination of Rhizosphere Soil Physicochemical Properties

Soil physicochemical properties were determined according to *Soil Agrochemical Analysis* [34]. The pH was measured using a PHS-2F pH meter (Leici Instrument Co., Ltd., Shanghai, China). Organic matter was determined by the potassium dichromate volumetric-external heating method. Total nitrogen was measured by the Kjeldahl method. Available nitrogen was determined by the alkaline hydrolysis diffusion method. Available phosphorus was extracted with sodium bicarbonate and measured by molybdenum-antimony anti-spectrophotometry. Available potassium was extracted with ammonium acetate and determined by atomic absorption flame photometry.

### 2.3. Analysis of the Microbial Community in the Tomato Rhizosphere Without Culture

#### 2.3.1. DNA Extraction and High-Throughput Sequencing

Total DNA extraction, amplification, library construction, and sequencing of rhizosphere soil and seedling substrate samples were completed by Guangdong Magigene Biotechnology Co., Ltd. (Shenzhen, China). Genomic DNA was extracted using the MOBIO PowerSoil^®^ DNA Isolation Kit (MO BIO Laboratories, Inc., Carlsbad, CA, USA). After quality verification with Nano Drop One (Thermo Fisher Scientific, Waltham, MA, USA), PCR amplification was performed using primers targeting the V4 region of bacterial 16S rRNA (515F/806R) and the ITS1 region of fungi (ITS5-1737F/ITS2-2043R) [35,36]. The 50 μL reaction mixture contained 25 μL 2× Premix Taq, 1 μL each of the forward and reverse primers, and approximately 50 ng of DNA template. The amplification program was 94 °C for 5 min; 30 cycles of 94 °C for 30 s, 52 °C for 30 s, and 72 °C for 30 s; and 72 °C for 10 min. Amplicons were detected by gel electrophoresis, purified, constructed into libraries, and sequenced on an Illumina NovaSeq 6000 platform with PE250 mode (Illumina, Inc., San Diego, CA, USA).

#### 2.3.2. Bioinformatic Analysis

Raw sequencing data were processed using the standard pipeline on the Magigene Cloud Platform. First, quality control and primer trimming were performed using fastp [37], followed by denoising and ASV (Amplicon Sequence Variant) clustering via the DADA2 algorithm [38]. Taxonomic annotation was carried out against the Silva 138 database (bacteria) and UNITE 8.0 database (fungi) to obtain ASV abundance tables and species composition profiles. Based on the ASV tables, the following analyses were conducted: (1) community composition: species composition and relative abundance at each taxonomic level; (2) alpha diversity: Shannon, Chao1, and Simpson indices to assess diversity, richness, and evenness; (3) beta diversity: principal coordinate analysis (PCoA) based on Bray–Curtis distance to evaluate community differences among samples; (4) differential species analysis: LEfSe (LDA > 4.0) to identify significantly different taxa among treatments and growth stages; (5) environment correlation: Spearman correlation between the top 10 genera and soil physicochemical properties (pH, organic matter, total nitrogen, available nitrogen, available phosphorus, and available potassium), with correlation heatmaps constructed.

### 2.4. Analysis of Culturable Bacterial Communities in the Tomato Rhizosphere

#### 2.4.1. Isolation and Purification of Strains

10 g of rhizosphere sample was added to a flask with 90 mL of sterile water and 10 sterile glass beads, then shaken at 180 rpm for 30 min to prepare a 10^−1^ suspension. A series of dilutions ranging from 10^−2^ to 10^−7^ were prepared using the 10-fold serial dilution method. 100 μL of dilutions at 10^−4^, 10^−5^, and 10^−6^ were spread onto beef extract peptone agar plates (beef extract 3 g/L, peptone 10 g/L, NaCl 5 g/L, agar 15 g/L) in triplicate [39]. After incubation at 28 °C for 36 h, colonies were counted with an automatic colony counter (Xunshu Technology, Wuhan, China). Single colonies were picked and purified by streaking based on morphology, color, and size. Purified strains were inoculated onto slant media and stored at 4 °C for further use.

#### 2.4.2. BOX-PCR Fingerprinting Analysis

Purified strains were cultured in beef extract peptone liquid medium (beef extract 3 g/L, peptone 10 g/L, NaCl 5 g/L) to the log phase. A 1 mL aliquot was centrifuged to collect cells, and genomic DNA was extracted using the Trelief^®^ Bacteria Genomic DNA Kit (Tsingke Biotechnology Co., Ltd., Beijing, China). PCR amplification was performed with the BOX A1R primer (5′-CTACGGCAAGGCGACGCTGACG-3′) [40]. The 20 μL mixture contained 2 μL of DNA, 0.5 μL of BOX A1R primer, 10 μL of 2×PCR Taq Mix, and ddH_2_O. The PCR program was 95 °C for 2 min; 30 cycles of 94 °C for 1 min, 52 °C for 1 min, and 65 °C for 8 min; and 65 °C for 16 min. Amplicons were separated by 2% agarose gel electrophoresis. Fingerprints were digitized with NTSYS software (version 2.10e), and a genetic similarity dendrogram was constructed using UPGMA clustering algorithm [41]. Representative strains from major clusters were selected for 16S rRNA gene amplification with universal primers 27F/1492R. PCR products were sequenced by Tsingke Biotechnology Co., Ltd. (Beijing, China) to clarify the species composition and distribution.

### 2.5. Screening of Rhizosphere Growth-Promoting Bacteria

#### 2.5.1. Determination of Phosphate-Solubilizing Capacity

Purified strains were spot-inoculated onto Pikovskaya (PKO) inorganic phosphorus solid medium [42]. After incubation at 28 °C for 5 d, transparent circles were observed. The diameters of transparent circles (D) and colonies (d) were measured to calculate the phosphate-solubilizing efficiency EC (D/d). Quantitative determination was performed using molybdenum-antimony anti-spectrophotometry [43]. Strains were incubated in PKO liquid medium at 28 °C and 120 rpm for 5 d. The fermentation broth was centrifuged at 8000 rpm for 10 min. The supernatant was diluted and mixed with a molybdenum–antimony chromogenic agent, and the absorbance was measured at 700 nm. The soluble phosphorus content was calculated based on the standard curve.

#### 2.5.2. Determination of Potassium-Solubilizing Capacity

Preliminary screening was carried out using Aleksanov medium [44]. After incubation at 28 °C for 5 d, transparent zones were observed. For quantitative analysis, strains forming transparent zones were cultured in Aleksanov liquid medium at 28 °C and 180 rpm for 3 d. The fermentation broth was centrifuged at 10,000 rpm for 10 min, and the potassium ion content in the supernatant was determined using a flame photometer [45].

#### 2.5.3. Determination of IAA-Producing Capacity

Strains were inoculated into a liquid medium containing 0.5 g/L L-tryptophan [46] and incubated at 28 °C and 180 rpm for 3 d. The culture broth was centrifuged at 8000 rpm for 5 min. Then, 2 mL of the supernatant was mixed with 4 mL of Salkowski reagent (50 mL of 35% HClO_4_ + 1 mL of 0.5 mol/L FeCl_3_), reacted in darkness for 30 min, and the absorbance was measured at 530 nm. The IAA yield was calculated based on the standard curve.

### 2.6. Pot Experiment

#### 2.6.1. Test Strains

Based on qualitative and quantitative screening results and 16S rRNA sequencing, four non-pathogenic strains were selected for pot experiments: S25095 (*Rouxiella badensis* subsp. *acadiensis*, with phosphate-solubilizing, potassium-solubilizing and IAA-producing abilities), S25101 (*Serratia marcescens*, phosphate-solubilizing), S25012 (*Priestia aryabhattai*, potassium-solubilizing), and S25041 (*Bacillus subtilis*, IAA-producing).

#### 2.6.2. Experimental Design

Six treatments were set, including five experimental groups and one control, with five replicates each. T1: inoculated with multifunctional strain S25095; T2: inoculated with a synthetic consortium (S25101, S25012, S25041); T3: inoculated with phosphate-solubilizing strain S25101; T4: inoculated with potassium-solubilizing strain S25012; T5: inoculated with IAA-producing strain S25041; CK: control without inoculation.

#### 2.6.3. Inoculant Preparation and Application

Strains were cultured in beef extract peptone liquid medium at 28 °C and 180 rpm to the log phase (OD_600_ = 0.8–1.0). Cells were harvested by centrifugation, resuspended in sterile saline, and adjusted to 1 × 10^8^ CFU/mL. The potting substrate consisted of a sterilized mixture of peat moss, perlite, and vermiculite. Each pot contained one tomato seedling. The plants were grown under laboratory conditions with artificial lighting (16 h light/8 h dark photoperiod) and were irrigated once per week with deionized water. At transplanting, 20 mL of the bacterial suspension was applied to each tomato seedling’s root.

#### 2.6.4. Determination of Related Indicators

Agronomic traits: At 60 days after inoculation, plant height, stem diameter, branch number, leaf number, leaf length, and leaf width were measured. Plant height was recorded from the base to the growing point; stem diameter with a vernier caliper; leaf number from the first green leaf to the top; and leaf length and width with a tape measure.

Root architecture: Root images were scanned and analyzed by WinRHIZO for total root length, surface area, average diameter, tip number, and fractal dimension.

Leaf enzyme activities and relative chlorophyll content (SPAD): Catalase (CAT), superoxide dismutase (SOD), peroxidase (POD), and ascorbate peroxidase (APX) were determined using microassay kits (Solarbio, Beijing). SPAD was measured with a SPAD-502PLUS chlorophyll meter (Xiangfan Instrument, Shanghai, China).

### 2.7. Data Analysis

Bioinformatics analysis of high-throughput sequencing was performed on the Magigene Cloud Platform. BOX-PCR fingerprinting was analyzed using NTSYSpc 2.1 (version 2.10e). Data were organized with Microsoft Excel 2019. One-way ANOVA (SPSS 25.0) and Duncan’s multiple range test (*p* < 0.05) were used to compare significant differences among treatments. Figures were plotted using Origin 2018 and GraphPad Prism 8.0.

## 3. Results

### 3.1. Rhizosphere Soil Physicochemical Properties

The physicochemical properties of tomato rhizosphere soil under different growth stages and seedling trays are shown in Table 2. The soil pH ranged from 6.10 to 6.41, which is slightly acidic. The soil organic matter (SOM) varied from 19.55 to 28.35 g/kg. The total nitrogen (TN) and available nitrogen (AN) increased with growth stages, with the highest values (1.71 g/kg and 149.25 mg/kg) in C.BM, which were higher than those in C.PM. The available phosphorus (AP) was generally higher in PM, reaching 82.18 mg/kg in B.PM. The available potassium (AK) was higher in BM, with C.BM showing the highest value, which was significantly higher than that in C.PM (*p* < 0.05).

### 3.2. Analysis of Uncultured Microbial Communities in Tomato Rhizosphere Soil and Nursery Substrate

#### 3.2.1. Microbial Community Structure

High-throughput sequencing was used to characterize the structure of uncultured microbial communities in the tomato rhizosphere under different growth stages and seedling trays. The results showed obvious differences in community composition among the treatments.

**Figure 1:**
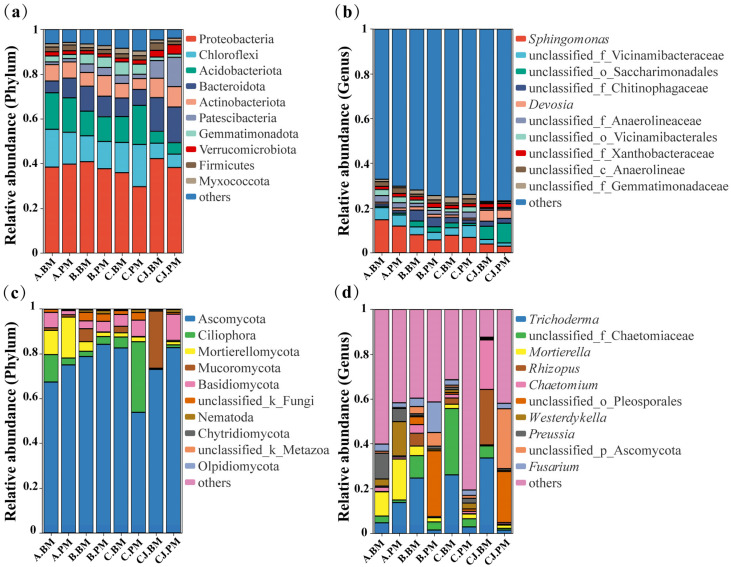
Composition of microbial communities in the tomato rhizosphere without culture: (**a**) relative abundance of bacterial community at the phylum level; (**b**) relative abundance of bacterial community at the genus level; (**c**) relative abundance of fungal community at the phylum level; (**d**) relative abundance of fungal community at the genus level.

At the phylum level (Figure 1a), Proteobacteria, Chloroflexi, Acidobacteriota, Bacteroidota, and Actinobacteriota were dominant in all samples, accounting for >70% relative abundance and forming the core bacterial community. Proteobacteria was the most abundant in both the rhizosphere soil and the seedling substrate. Chloroflexi and Acidobacteriota were mainly enriched in the rhizosphere soil, while Bacteroidota was more abundant in the seedling substrate. At the genus level (Figure 1b), *Sphingomonas* was the most dominant annotated genus. It was significantly enriched in A.BM (14.76%) and A.PM (11.93%) but lowest in the seedling substrate (CJ.BM, CJ.PM). *Devosia* was more abundant in the substrate (CJ.BM: 4.87%, CJ.PM: 3.75%) than in the rhizosphere soils (0.71–1.48%). Unclassified_o_Saccharimonadales showed a similar distribution pattern to *Devosia*.

For fungal communities, at the phylum level (Figure 1c), Ascomycota was the dominant phylum, with a relative abundance exceeding 50% in all treatments, followed by Ciliophora and Mortierellomycota. The relative abundance of Ascomycota increased with growth stages in BM rhizosphere soil, while it first increased and then decreased in PM soil. Ciliophora and Mortierellomycota were mainly distributed in the rhizosphere soil and were rare in the seedling substrate. At the genus level (Figure 1d), distinct differences were observed among treatments. *Trichoderma* was the most abundant annotated genus and was significantly enriched under the BM treatment. Its relative abundance reached 24.68%, 26.11%, and 33.65% in B.BM, C.BM, and CJ.BM, respectively, but was below 2.83% in all PM treatments. In addition, *Fusarium*, an important soil-borne pathogen, showed the highest abundance in B.PM (13.67%) and the lowest in CJ.BM (0.22%).

#### 3.2.2. Alpha Diversity Analysis

**Figure 2:**
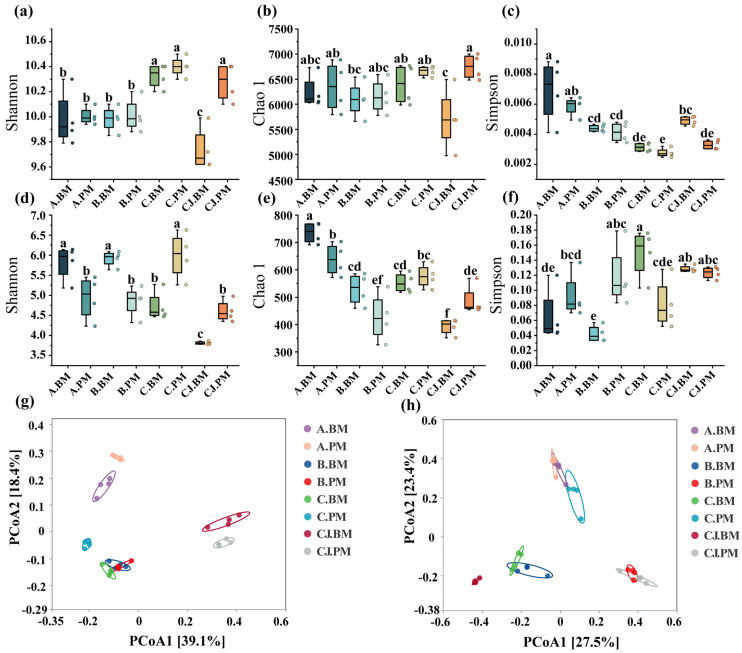
Alpha and beta diversity of tomato rhizosphere microbial communities: (**a**) bacterial Shannon index; (**b**) bacterial Chao1 index; (**c**) bacterial Simpson index; (**d**) fungal Shannon index; (**e**) fungal Chao1 index; (**f**) fungal Simpson index; (**g**) bacterial beta diversity; (**h**) fungal beta diversity. Different lowercase letters above the boxplots indicate significant differences among treatments at *p* < 0.05.

The Shannon, Chao1, and Simpson indices were used to evaluate diversity, richness, and evenness (Figure 2a–f).

The bacterial Shannon index ranged from 9.74 to 10.40, with the highest values at the peak fruiting stage: 10.33 (C.BM) and 10.40 (C.PM). Chao1 varied from 5710.8 to 6749.0, showing a similar trend to Shannon. A lower Simpson index indicates higher evenness. C.BM (0.00314) and C.PM (0.00277) showed the highest evenness. The seedling stage had the highest Simpson index (0.00690 in A.BM), significantly higher than the other treatments (*p* < 0.05), indicating lower evenness and a higher concentration.

For fungal communities, the Shannon index ranged from 3.81 to 6.04. At the seedling and early fruiting stages, the Shannon index was significantly higher in BM than in PM (*p* < 0.05). The Chao1 index was highest at the seedling stage (735.18 in A.BM, 638.55 in A.PM) and lowest in the peak fruiting stage substrate (392.33 in CJ.BM, 487.40 in CJ.PM). The Simpson index varied from 0.042 to 0.149. B.BM showed the lowest Simpson index, significantly lower than B.PM (*p* < 0.05), indicating higher fungal evenness under BM at the early fruiting stage. The opposite trend occurred at the peak fruiting stage, with higher dominance and lower evenness under BM.

#### 3.2.3. Beta Diversity Analysis

Principal coordinate analysis (PCoA) based on the Bray–Curtis distance matrix was performed to clarify the effects of treatments and growth stages on the rhizosphere microbial community structure. Closer points indicate more similar communities, while dispersed points indicate greater differences.

For bacteria (Figure 2g), PCoA1 and PCoA2 explained 39.1% and 18.4% of the total variation, respectively. The rhizosphere soil at the seedling stage (A.BM, A.PM), early and peak fruiting stage rhizosphere soils (B.BM, B.PM, C.BM, C.PM), and peak fruiting stage substrate (CJ.BM, CJ.PM) formed three distinct clusters, revealing significant differences in bacterial community structure. The growth stage had a stronger effect than the seedling tray treatment.

For fungi (Figure 2h), PCoA1 and PCoA2 explained 27.5% and 23.4% of the total variation (cumulative 50.9%). Except for the seedling-stage soil (A.BM, A.PM), samples from the same growth stage but different trays were clearly separated.

#### 3.2.4. Species Difference Analysis

The LEfSe analysis was used to identify indicator taxa and enrichment characteristics of rhizosphere microbial communities under different seedling trays and growth stages. The LDA threshold was set at 4.0 for bacteria and fungi, with taxonomic levels from phylum to genus.

**Figure 3:**
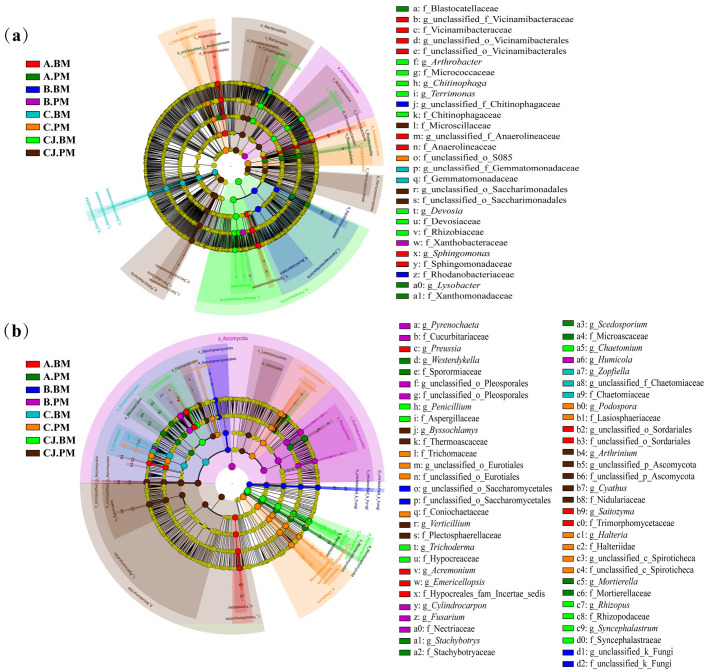
Indicator species and composition: (**a**) bacterial indicator species and enrichment characteristics; (**b**) fungal indicator species and enrichment characteristics.

In bacterial communities, 64 significantly different taxa were identified from phylum to genus. The evolutionary cladogram showed representative taxa at the family and genus levels (Figure 3a); the LDA bar plot is shown in Figure S1. The main indicator bacteria were *Sphingomonas* (A.BM) and Xanthomonadaceae (A.PM) at the seedling stage; Gammaproteobacteria (B.BM) and Actinobacteriota (B.PM) at the early fruiting stage; Gemmatimonadota (C.BM) and Chloroflexi (C.PM) at the peak fruiting stage; and Alphaproteobacteria (CJ.BM) and Patescibacteria (CJ.PM) in the peak fruiting stage substrate.

For fungi, LEfSe analysis identified 94 significantly different taxa from phylum to genus. The cladogram (Figure 3b) showed representative taxa at the family and genus levels; the LDA bar plot is shown in Figure S2. With the progression of growth stages, the main indicator fungi were *Preussia* (A.BM), Saccharomycetales (B.BM), Sordariomycetes (C.BM), and *Trichoderma* (CJ.BM) under BM; and Mortierellales (A.PM), Dothideomycetes (B.PM), Ciliophora (C.PM), and Agaricomycetes (CJ.PM) under PM. Most of the enriched fungi were beneficial, but Figure S2 revealed pathogens, including *Fusarium* and *Pyrenochaeta* in B.PM, that commonly cause root rot and stem rot.

#### 3.2.5. Environmental Factor Correlation Analysis

For this analysis, only rhizosphere soil samples (A.BM, A.PM, B.BM, B.PM, C.BM, C.PM) were included, as physicochemical data were not measured for the nursery substrate samples (CJ.BM and CJ.PM). The top 10 bacterial genera and top 10 fungal genera based on relative abundance in these soil samples were selected for Spearman correlation analysis with soil properties.

**Figure 4:**
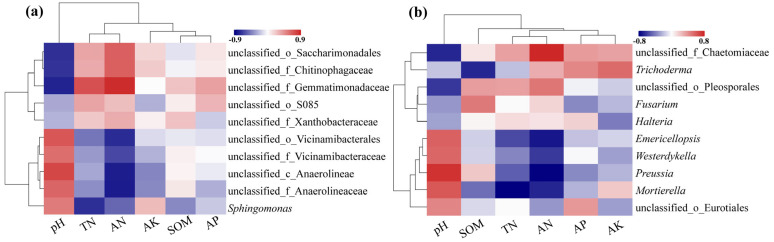
Correlation between microbial communities and environmental factors: (**a**) correlation between bacterial genera and soil properties; (**b**) correlation between fungal genera and soil properties.

The results (Figure 4a) showed that pH and available nitrogen (AN) were key driving factors. Unclassified_f_Gemmatimonadaceae was negatively correlated with pH (R = −0.78) and positively correlated with AN (R = 0.87). Unclassified_c_Anaerolineae was positively correlated with pH (R = 0.76) and negatively correlated with AN (R = −0.80).

For fungal communities (Figure 4b), pH and AN were also dominant drivers. *Preussia* was positively correlated with pH (R = 0.75) and negatively correlated with AN (R = −0.79). In contrast, Unclassified_f_Chaetomiaceae was negatively correlated with pH (R = −0.68) and positively correlated with AN (R = 0.81).

### 3.3. Analysis of Cultivable Bacterial Communities in Tomato Rhizosphere Soil and Nursery Substrate

#### 3.3.1. Rhizosphere Bacterial Enumeration and Isolation Results

Bacterial colonies were cultured using the pour plate method for 36 h. An automatic colony counter was used to determine cultivable microbial counts (CFU/g) in the rhizosphere soil and substrate under different treatments and growth stages (Table 3).

In the BM group, the microbial count was highest at the seedling stage (A.BM: 2.53 × 10^9^ CFU/g) and decreased significantly with the growth stage, reaching 4.46 × 10^7^ CFU/g at the early fruiting stage (B.BM) and 9.30 × 10^6^ CFU/g at the peak fruiting stage (C.BM). The PM group showed a similar trend. Moreover, at the same growth stage, microbial counts were higher under BM than PM in both the rhizosphere soil and seedling substrate.

After counting, 106 bacterial strains were isolated and purified from the rhizosphere soil and seedling substrate across different growth stages and seedling tray treatments based on colony morphological characteristics (size, color, shape, etc.). Among them, 59 strains were isolated from the BM group and 47 from the PM group (Table 4).

#### 3.3.2. BOX-PCR Fingerprinting Analysis

BOX primers were used to amplify 106 purified strains to obtain fingerprint profiles. Bands at the same position were scored as “1” and absent bands as “0”. Clustering was performed using the unweighted pair-group method with arithmetic mean (UPGMA) in NTSYS-pc 2.1 to construct a dendrogram.

**Figure 5:**
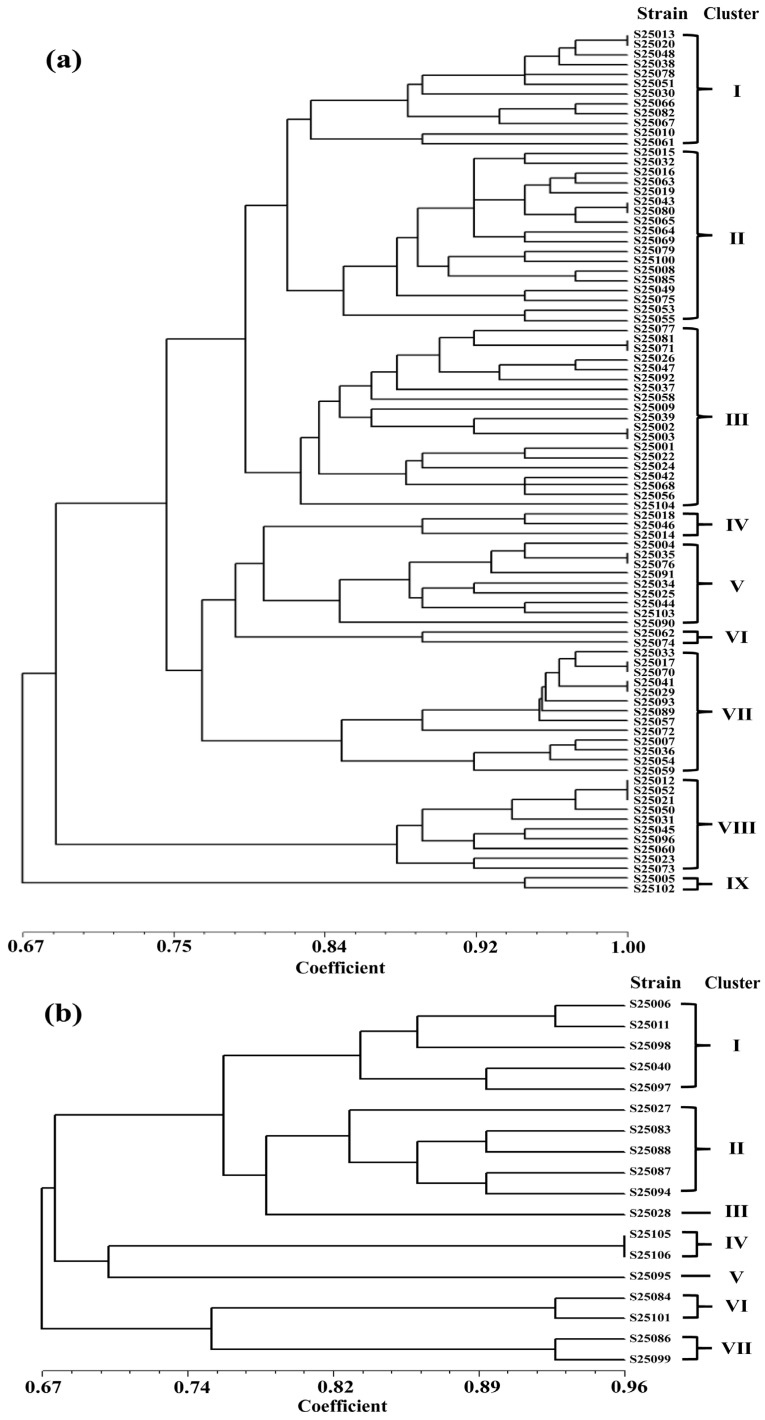
Dendrogram of genetic similarity of cultivable bacteria from tomato rhizosphere based on BOX-PCR fingerprinting: (**a**) Gram-positive bacteria; (**b**) Gram-negative bacteria.

As shown in Figure 5a, 88 Gram-positive strains were grouped into nine clusters at 82% similarity. Cluster III contained the most strains (19), followed by Cluster II (18) and Cluster VII (13). Representative strains of each cluster and their closest relatives are listed in Table 5. Strain S25037 (Cluster III) showed 100% similarity to *Bacillus zanthoxyli* (OL875277.1); S25063 (Cluster II) showed 99.93% similarity to *Bacillus siamensis* (MN176482.1); S25089 (Cluster VII) showed 100% similarity to *Bacillus subtilis* (MK267098.1).

In the Gram-negative bacterial dendrogram (Figure 5b), 18 strains formed 7 clusters at 82% similarity. Clusters I and II contained the most strains. Representative strains of each cluster and their closest relatives are shown in Table 6. Strain S25098 (Cluster I) shared 100% similarity with *Brucella anthropi* (MH281752.1); S25088 (Cluster II) showed 99.78% similarity to *Chryseobacterium* sp. (OR496587.1). Clusters III and V had the fewest strains. Strain S25028 (Cluster III) showed 98.76% similarity to *Pseudomonas oryzagri* (MT514506.1). Cluster V consisted of only one strain, S25095 (from CJ.BM), with 99.78% similarity to *Rouxiella badensis* subsp. *acadiensis*. (CP060592.1).

### 3.4. Screening Results of Growth-Promoting Bacteria from Rhizosphere Soil and Nursery Substrate

Culture-independent and cultivable microbial analysis showed that BM treatment favored beneficial microbial enrichment, while PM treatment tended to accumulate potential pathogens. Based on this difference, 106 isolated bacterial strains were screened for phosphate solubilization, potassium solubilization, and IAA production to compare their growth-promoting abilities across different sources.

#### 3.4.1. Screening Results of Phosphate-Solubilizing Bacteria

1. Qualitative screening

**Figure 6:**
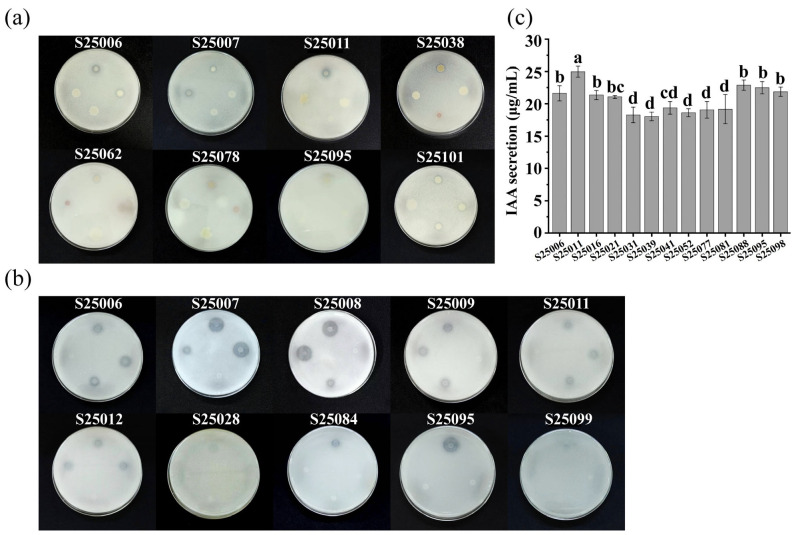
Screening results of rhizosphere growth-promoting bacteria: (**a**) phosphate-solubilizing strains (numbers correspond to the top strains on the plate); (**b**) potassium-solubilizing strains (numbers correspond to the top strains on the plate); (**c**) IAA secretion of tested strains. Different lowercase letters on the error bars indicate significant differences among treatments at *p* < 0.05.

Strains were spotted on PKO inorganic phosphorus medium and cultured for 5 days. Eight phosphate-solubilizing strains were screened (Figure 6a). Phosphate solubilization was determined by halo zone formation, with the EC ratio (D/d, halo zone diameter/colony diameter) as the index. Table 7 shows the following: ① 62.5% of the eight strains were from the BM treatment; ② colony diameters ranged from 3.5 to 11.89 mm, and halo zone diameters ranged from 7.75 to 13.2 mm. Strain S25006 (from the BM treatment) had the highest EC value (D/d = 2.51).

2. Quantitative screening

Phosphate solubilization was quantified by molybdenum-antimony anti-spectrophotometry with pH measurement. Table 7 shows the following: ① the highest soluble phosphorus was 206.47 mg/L (S25011) from the BM treatment at the seedling stage, followed by 195.52 mg/L (S25006), also from the BM treatment; ② efficient strains showed a pH decline. Strain S25095 from the BM treatment at the peak fruiting stage had the largest pH drop (ΔpH = 1.95).

#### 3.4.2. Screening Results of Potassium-Solubilizing Bacteria

1. Qualitative screening

After 5 days of incubation on Aleksandrov solid medium, 10 potassium-solubilizing strains were screened based on halo zone formation (Figure 6b). Table 8 shows: ① 60% of the 10 strains were from the BM treatment at the seedling stage; ② Strains S25007, S25008, and S25095 from the BM treatment exhibited strong potassium-solubilizing ability, with D/d values of 3.35, 3.46, and 3.35, respectively (D/d: ratio of halo zone diameter to colony diameter).

2. Quantitative screening

The 10 strains were cultured in Aleksandrov liquid medium for 3 days. Potassium ion concentration in the supernatant was determined by flame photometry. Results (Table 8) showed: ① The highest soluble potassium (2.35 mg/L) was observed in strains S25008 and S25012 from the BM treatment; ② The average soluble potassium of strains from the BM treatment was higher than that from the PM treatment.

#### 3.4.3. Screening Results of IAA-Producing Bacteria

Indole-3-acetic acid (IAA) is a key hormone promoting plant growth. In this study, 13 IAA-producing bacterial strains were screened using IAA selection liquid medium containing 0.5 g/L L-tryptophan (Figure 6c).

Approximately 61.5% of the IAA-producing strains were from the BM treatment. The IAA yields of these 13 strains ranged from 18.03 to 24.98 μg/mL. The highest yield was observed in strain S25011 (24.98 μg/mL), followed by S25088 (22.87 μg/mL), both from the BM treatment.

### 3.5. Pot Experiment Results

#### 3.5.1. Agronomic Traits of Tomato Under Different Treatments

**Figure 7:**
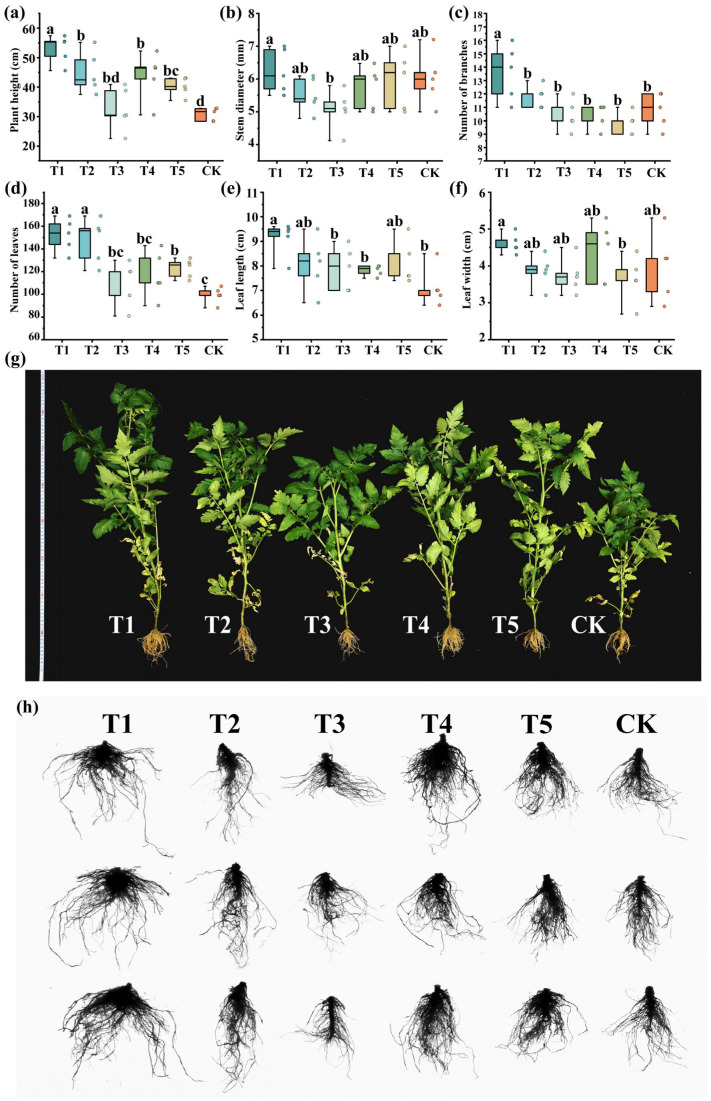
Agronomic traits and roots of tomato under different treatments: (**a**) plant height; (**b**) stem diameter; (**c**) number of branches; (**d**) number of leaves; (**e**) leaf length; (**f**) leaf width; (**g**) photos of tomato plants; (**h**) root scanning results. Different lowercase letters above the boxplots indicate significant differences among treatments at *p* < 0.05.

Plant height, stem diameter, branch number, leaf number, leaf length, and leaf width were measured 60 days after inoculation. Inoculation with growth-promoting bacteria significantly promoted tomato growth (Figure 7). The highest plant heights were observed in T1 (a multifunctional strain) and T2 (a synthetic community), reaching 52.86 cm and 45.06 cm, respectively, which were significantly higher than CK (*p* < 0.05), with increases of 71.40% and 46.11%. Stem diameter varied slightly among treatments. T1 had the most branches (13.6), significantly higher than the other treatments and CK (*p* < 0.05), with a 25.9% increase. T1 and T2 had the most leaves (152.2 and 147.2), 53.4% and 48.4% higher than CK. Leaf length was greatest in T1 (9.12 cm), significantly higher than T3, T4, and CK (*p* < 0.05). Leaf width showed little difference, but T1 (4.60 cm) was the maximum.

#### 3.5.2. Root Architecture Under Different Treatments

Total root length, root tip number, root diameter, root surface area, and root fractal dimension were determined by a root scanner to evaluate the effects of different PGPR strains and their combinations on tomato root development (Table 9, Figure 7h). The total root length ranged from 347.03 to 634.37 cm, with T1 (a multifunctional strain) reaching the maximum (634.37 cm), significantly higher than CK and other treatments (*p* < 0.05). The root tip number, an important index of root branching and absorption potential, was highest in T1 (316.33), followed by T2 (232.33). The root diameter was largest in T1 (1.86 mm) and T4 (1.83 mm), indicating promotion of root thickening. The root surface area showed a similar trend to the total root length, with T1 having the maximum (65.60 cm^2^), significantly higher than CK and other treatments (*p* < 0.05). The root fractal dimension ranged from 1.64 to 1.73, with T2 (1.73) and T1 (1.72) being the highest.

#### 3.5.3. Leaf Physiological Indices Under Different Treatments

**Figure 8:**
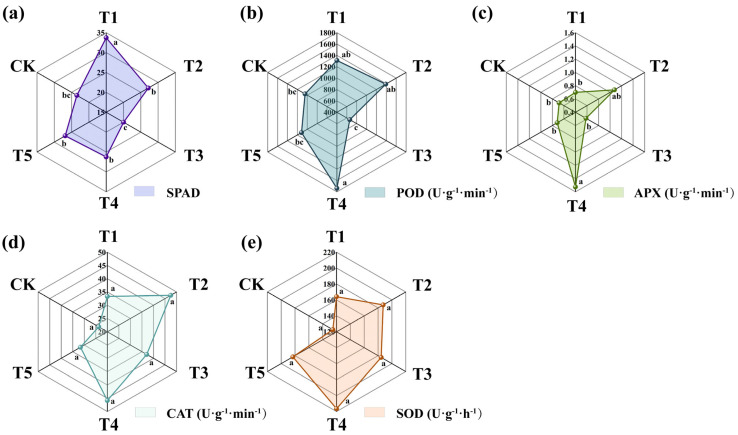
Physiological indices of tomato leaves under different treatments: (**a**) relative chlorophyll content; (**b**) peroxidase; (**c**) ascorbate peroxidase; (**d**) catalase; (**e**) superoxide dismutase. Different lowercase letters indicate significant differences among treatments at *p* < 0.05.

To investigate the effects of different PGPR strains and their combinations on tomato leaf physiological function, the relative chlorophyll content (SPAD) and antioxidant enzyme activities, including peroxidase (POD), ascorbate peroxidase (APX), catalase (CAT), and superoxide dismutase (SOD), were measured 60 days after pot cultivation. The results (Figure 8) showed that T1 had the highest SPAD value (33.72), significantly higher than the other treatments and CK (*p* < 0.05), followed by T2 (27.18). The activities of POD and APX were highest in T4, reaching 1738.89 U/g and 1.53 U/g, respectively, which were 66.49% and 123.43% higher than those in CK (*p* < 0.05). The activities of CAT and SOD showed no significant differences among treatments but were higher in all inoculated treatments than in CK.

## 4. Discussion

This study used rhizosphere soil and nursery substrate of tomato at different growth stages under biomass and plastic tray nursery systems to clarify the effects of different treatments on soil physicochemical properties and microbial community structure. A highly efficient multifunctional PGPR strain represented by S25095 was screened, providing theoretical support and high-quality microbial resources for tomato cultivation.

Soil physicochemical properties are critical for shaping microbial community structure. In this study, the TN and AN contents in tomato rhizosphere soil increased continuously with growth stage, likely due to enhanced root exudates promoting organic nitrogen mineralization and accumulation via the rhizosphere priming effect [47]. Notably, the BM treatment not only increased soil total nitrogen and available nitrogen content, but also significantly enhanced available potassium accumulation, whereas the PM treatment showed higher available phosphorus accumulation. This difference in nutrient distribution may be related to the degradation characteristics of the two tray materials. The BM trays slowly release organic matter and potassium during degradation. Indeed, it has been reported that the application of biochar-based materials can increase soil available potassium content by 72.83% [48]. A limitation of this study is that the biomass seedling tray simultaneously introduced both physical structural changes and additional nutrients. Therefore, the observed positive effects on soil physicochemical properties cannot be solely attributed to the tray material itself. Nevertheless, our primary objective was to evaluate whether biomass seedling trays could serve as a practical alternative to conventional plastic trays under real agricultural conditions. Future studies should include a control group consisting of plastic trays supplemented with equivalent nutrients to decouple the physical and chemical effects of biomass trays. Spearman correlation analysis showed that pH and AN significantly regulated both bacterial and fungal community structures, consistent with findings in most Solanaceae crops [49,50,51], highlighting the role of basic soil properties in rhizosphere microbial assembly.

Culture-independent analysis of tomato rhizosphere microbial communities revealed that BM treatment favored the construction of a healthy rhizosphere microecology. BM significantly enriched beneficial fungi such as *Trichoderma*, whereas PM accumulated soil-borne pathogens, including *Fusarium* and *Pyrenochaeta*, suggesting that biomass nursery materials can recruit beneficial microbes and suppress pathogens. Li et al. reported that biomass trays possess favorable porosity for aeration, water exchange, and nutrient cycling, providing an ideal niche for beneficial rhizosphere colonization [52]. The enrichment of *Trichoderma* in the BM treatment is particularly noteworthy, as this genus is well-known for its multiple biocontrol and plant growth-promoting activities. *Trichoderma* species antagonize phytopathogens through several complementary mechanisms, including mycoparasitism, antibiosis via the production of bioactive secondary metabolites, competition for space and nutrients, and the induction of systemic resistance in host plants [53,54]. It is noteworthy that Diatta et al. demonstrated that the combined use of fish effluent and organic manure enhanced crop growth and yield through continuous nutrient supply [55]. In line with this finding, the significant enrichment of *Trichoderma* in the BM treatment in this study may be attributed not only to the physical niche provided by the biomass trays but also to their slow-release nutrient properties. Adequate nutrient availability may have enhanced the metabolic activity of *Trichoderma* and its ability to synthesize biocontrol-related secondary metabolites, thereby more effectively suppressing soil-borne pathogens. Additionally, numerous unclassified taxa (e.g., unclassified_o_Saccharimonadales and unclassified_f_Gemmatimonadaceae) were detected, reflecting the unique and exploitable microbial resources in the dry-hot valley. As reported by Rinke et al., such uncultured “microbial dark matter” often harbors undescribed metabolic functions [56].

Analysis of the genetic diversity of culturable bacteria is also vital for exploring microbial resources [57,58]. Using BOX-PCR fingerprinting, this study further clarified the effects of different treatments on the genetic diversity and dominant functional groups of culturable rhizosphere bacteria in tomato. The BM treatment provided a more suitable habitat, with significantly higher colony counts than PM. This agrees with Dukare et al., who reported that biomass mulch increased culturable fungi, actinomycetes, and functional microbes in the tomato rhizosphere by releasing carbon sources and improving microhabitats, whereas plastic restricted microbial growth [59]. A distinct multifunctional strain, S25095 (*Rouxiella badensis* subsp. *acadiensis*), was identified, demonstrating the advantage of BM in mining rhizosphere microbial resources.

In the screening of plant growth-promoting bacteria, the most effective strains originated from BM treatment and performed better in soluble P, soluble K, and IAA production. This indicates that a healthy and stable rhizosphere favors the survival and enrichment of functional PGPR. Although the PM treatment harbored fewer and weaker PGPR, several excellent strains were still obtained. This suggests that the nursery method is not the sole determinant of strain growth-promoting function. Pot experiments further confirmed that the multifunctional strain S25095 (treatment T1) from BM achieved the best growth promotion, significantly improving both aboveground and underground tomato growth. The synthetic community T2 did not outperform T1, likely due to interstrain competition reducing rhizosphere colonization efficiency and weakening overall function; simple functional stacking does not equal a synergistic effect. Zhao et al. found that the wild-type *Pseudomonas protegens* inhibited *Bacillus velezensis* via pyoluteorin, resulting in lower biofilm formation, colonization, and biocontrol than a single *Bacillus velezensis*. Synergy occurred only after knocking out the pyoluteorin synthesis gene [60]. Xu et al. also noted in a systematic review that bottom-up synthetic communities frequently show antagonism due to poor compatibility, while single multifunctional strains exhibit higher colonization stability and functional efficiency without interspecific competition [61]. Besides direct antagonism among strains, the poor performance of the synthetic community may also be attributed to resource competition caused by niche overlap. When multiple functional strains are co-inoculated, they may compete for the same carbon sources and colonization sites in the rhizosphere, thereby weakening their respective growth-promoting efficiency. In fact, it has been reported that inoculation with synthetic microbial communities can increase niche overlap by 59.2–62.4%, thereby exacerbating competition among community members [62]. Therefore, future construction of synthetic communities should prioritize ecological compatibility among strains rather than simply pursuing functional diversity [63,64]. In addition, inoculation enhanced leaf antioxidant enzyme activities, consistent with Rahman et al. and Lucas et al., who verified that PGPR significantly improved antioxidant enzyme activities in tomato [65,66], supporting the core mechanism proposed by Gowtham et al. that PGPR enhance plant stress resistance by inducing the antioxidant defense system [67]. Therefore, PGPR promote plant growth and strengthen stress resistance, providing reliable microbial resources for stress-tolerant tomato cultivation.

## 5. Conclusions

This study systematically investigated the effects of different nursery tray treatments on rhizosphere microbial communities of tomato in the dry-hot valley and screened and functionally verified efficient plant growth-promoting bacteria across all treatments. The results demonstrated that soil pH and AN were key abiotic factors driving rhizosphere microbial community assembly. The biomass nursery trays optimized the rhizosphere microenvironment, significantly enriched beneficial functional microorganisms, and reduced the relative abundance of soil-borne pathogens, indicating their potential to favorably modulate the rhizosphere microbial community structure. Similarly, BOX-PCR genetic diversity analysis revealed that the BM treatment enriched numerous beneficial taxa represented by *Bacillus*. In the subsequent growth-promoting bacteria screening, strains derived from the BM treatment exhibited stronger overall growth-promoting performance. Among them, the multifunctional strain S25095 significantly improved tomato agronomic traits and root architecture, with superior growth-promoting effects compared to single-function strains and synthetic microbial communities. This study provides microecological regulation strategies and high-quality microbial resources for tomato cultivation in the dry-hot valley. However, the field colonization characteristics of the strain and the interaction mechanism between biomass materials and microorganisms still require further research in order to lay a theoretical foundation for the development of high-efficiency microbial inoculants.

## Figures and Tables

**Table 1:** Information of tested samples.

Sample ID	Growth Stage	Seedling Tray Type	Sample Type
A.BM	Seedling stage A	Biomass seedling tray	Rhizosphere soil
A.PM	Seedling stage A	Plastic seedling tray	Rhizosphere soil
B.BM	Early fruiting stage B	Biomass seedling tray	Rhizosphere soil
B.PM	Early fruiting stage B	Plastic seedling tray	Rhizosphere soil
C.BM	Peak fruiting stage C	Biomass seedling tray	Rhizosphere soil
C.PM	Peak fruiting stage C	Plastic seedling tray	Rhizosphere soil
CJ.BM	Peak fruiting stage C	Biomass seedling tray	Seedling substrate
CJ.PM	Peak fruiting stage C	Plastic seedling tray	Seedling substrate

**Table 2:** Physicochemical properties of rhizosphere soil under different treatments.

Sample ID	pH	SOM (g/kg)	TN (g/kg)	AN (mg/kg)	AP (mg/kg)	AK (mg/kg)
A.BM	6.28 ± 0.05 b	23.59 ± 1.77 b	1.45 ± 0.03 b	97.87 ± 7.01 c	52.96 ± 2.12 b	212.16 ± 30.91 ab
A.PM	6.41 ± 0.05 a	20.3 ± 1.09 c	1.46 ± 0.03 b	93.31 ± 4.04 c	58.99 ± 1.95 a	212.91 ± 19.30 ab
B.BM	6.1 ± 0.04 c	19.55 ± 1.00 c	1.47 ± 0.06 b	117.75 ± 5.32 b	73.69 ± 6.46 a	220.58 ± 23.16 a
B.PM	6.13 ± 0.04 c	28.35 ± 0.40 a	1.67 ± 0.05 a	116.55 ± 3.98 b	82.18 ± 4.48 b	201.21 ± 12.56 ab
C.BM	6.13 ± 0.01 c	22.89 ± 0.20 b	1.71 ± 0.04 a	149.25 ± 4.06 a	72.30 ± 11.55 a	232.25 ± 17.15 a
C.PM	6.13 ± 0.05 c	23.3 ± 1.28 b	1.66 ± 0.10 a	114.21 ± 4.10 b	81.40 ± 8.40 a	179.54 ± 11.88 b

Note: Values are mean ± standard deviation. Different letters in the same column indicate significant differences among treatments at *p* < 0.05.

**Table 3:** Number of culturable microorganisms per gram of soil and substrate.

Sample ID	Number of Culturable Microorganisms (CFU/g)
A.BM	2.53 × 10^9^ ± 8.90 × 10^8^ a
A.PM	4.53 × 10^8^ ± 4.74 × 10^8^ b
B.BM	4.46 × 10^7^ ± 1.49 × 10^7^ b
B.PM	3.47 × 10^7^ ± 1.45 × 10^7^ b
C.BM	9.30 × 10^6^ ± 5.00 × 10^5^ b
C.PM	1.26 × 10^7^ ± 2.52 × 10^5^ b
CJ.BM	2.46 × 10^7^ ± 5.57 × 10^5^ b
CJ.PM	1.94 × 10^7^ ± 6.56 × 10^5^ b

Note: Values are mean ± standard deviation. Different letters in the same column indicate significant differences among treatments at *p* < 0.05.

**Table 4:** Isolation results of rhizosphere strains.

Sample ID	Number of Strains	Strain ID
A.BM	18	S25001-S25018
A.PM	16	S25019-S25034
B.BM	16	S25035-S25050
B.PM	14	S25051-S25064
C.BM	9	S25065-S25073
C.PM	6	S25074-S25079
CJ.BM	16	S25080-S25095
CJ.PM	11	S25096-S25106

**Table 5:** Gram-positive representative strains and identification of their related species.

Cluster	Number of Strains	Representative Strain	Related Strain	Similarity
I	12	S25048	*Bacillus velezensis* (MZ276294.1)	100%
II	18	S25063	*Bacillus siamensis* (MN176482.1)	99.93%
III	19	S25037	*Bacillus zanthoxyli* (OL875277.1)	100%
IV	3	S25018	*Bacillus tequilensis* (MN543830.1)	100%
V	9	S25034	*Bacillus safensis* (NR_113945.1)	100%
VI	2	S25062	*Bacillus altitudinis* (OQ618976.1)	100%
VII	13	S25089	*Bacillus subtilis* (MK267098.1)	100%
VIII	10	S25023	*Priestia aryabhattai* (PV810461.1)	100%
IX	2	S25005	*Microbacterium paraoxydans* (LT629770.1)	99.93%

**Table 6:** Gram-negative representative strains and identification of their related species.

Cluster	Number of Strains	Representative Strain	Related Strain	Similarity
I	5	S25098	*Brucella anthropi* (MH281752.1)	100%
II	5	S25088	*Chryseobacterium* sp. (OR496587.1)	99.78%
III	1	S25028	*Pseudomonas oryzagri* (MT514506.1)	98.76%
IV	2	S25106	*Cupriavidus necator* (MN117667.1)	99.55%
V	1	S25095	*Rouxiella badensis* subsp. *acadiensis* (CP060592.1)	99.78%
VI	2	S25101	*Serratia marcescens* (CP139958.1)	99.64%
VII	2	S25099	*Serratia sarumanii* (CP124750.1)	99.64%

**Table 7:** Relevant indicators for screening phosphate-solubilizing bacteria.

Strain ID	Halo Zone Diameter (D)/mm	Colony Diameter (d)/mm	EC Value (D/d)	Soluble Phosphorus/mg/L	Initial pH	pH After 5 d	ΔpH
S25006	8.50 ± 0.80 ^b^	3.50 ± 0.58 ^d^	2.51 ± 0.70 ^a^	195.52 ± 4.52 ^b^	6.80 ± 0.40 ^a^	4.94 ± 0.40 ^bcd^	1.86 ± 0.77 ^a^
S25007	8.00 ± 0.77 ^b^	7.00 ± 0.67 ^bc^	1.14 ± 0.01 ^c^	18.82 ± 1.60 ^fg^	6.98 ± 0.31 ^a^	6.14 ± 0.51 ^a^	0.84 ± 0.47 ^b^
S25011	7.75 ± 0.76 ^b^	4.00 ± 0.59 ^d^	1.95 ± 0.09 ^b^	206.47 ± 4.18 ^a^	5.95 ± 0.29 ^bc^	4.39 ± 0.38 ^d^	1.56 ± 0.09 ^a^
S25038	9.20 ± 0.85 ^b^	8.00 ± 0.75 ^b^	1.15 ± 0.00 ^c^	52.04 ± 2.18 ^c^	6.03 ± 0.37 ^bc^	5.29 ± 0.49 ^abc^	0.74 ± 0.29 ^b^
S25062	7.75 ± 0.78 ^b^	6.55 ± 0.64 ^c^	1.18 ± 0.03 ^c^	28.47 ± 1.56 ^e^	5.60 ± 0.31 ^c^	4.73 ± 0.42 ^cd^	0.87 ± 0.12 ^b^
S25078	8.80 ± 0.89 ^b^	8.19 ± 0.82 ^b^	1.07 ± 0.01 ^c^	36.33 ± 2.05 ^d^	5.97 ± 0.31 ^bc^	5.61 ± 0.54 ^abc^	0.36 ± 0.23 ^b^
S25095	13.20 ± 1.05 ^a^	11.89 ± 0.86 ^a^	1.11 ± 0.02 ^c^	15.34 ± 1.64 ^g^	6.75 ± 0.33 ^a^	4.80 ± 0.44 ^cd^	1.95 ± 0.14 ^a^
S25101	8.50 ± 0.73 ^b^	7.95 ± 0.74 ^b^	1.07 ± 0.01 ^c^	23.31 ± 1.89 ^f^	6.42 ± 0.29 ^ab^	5.71 ± 0.55 ^ab^	0.71 ± 0.39 ^b^

Note: Values are mean ± standard deviation. Different letters in the same column indicate significant differences among treatments at *p* < 0.05.

**Table 8:** Related indicators of screening potassium-solubilizing bacteria.

Strain ID	Halo Zone Diameter (D)/mm	Colony Diameter (d)/mm	EC Value (D/d)	Supernatant K^+^ Concentration/mg/L
S25006	10.20 ± 2.27 ^b^	4.15 ± 0.89 ^a^	2.60 ± 0.99 ^ab^	1.75 ± 0.31 ^abc^
S25007	14.79 ± 1.94 ^a^	4.59 ± 0.97 ^a^	3.35 ± 0.89 ^a^	1.70 ± 0.37 ^abc^
S25008	14.49 ± 2.25 ^a^	4.35 ± 0.89 ^a^	3.46 ± 0.92 ^a^	2.35 ± 0.45 ^a^
S25009	8.39 ± 1.95 ^bcd^	4.35 ± 0.95 ^a^	2.05 ± 0.85 ^b^	1.15 ± 0.33 ^c^
S25011	7.90 ± 2.32 ^bcde^	4.25 ± 0.86 ^a^	1.95 ± 0.65 ^b^	1.45 ± 0.36 ^bc^
S25012	8.56 ± 2.14 ^bc^	4.54 ± 0.81 ^a^	1.89 ± 0.07 ^b^	2.35 ± 0.44 ^a^
S25028	5.90 ± 1.93 ^e^	4.62 ± 1.13 ^a^	1.36 ± 0.52 ^b^	1.25 ± 0.30 ^c^
S25084	6.85 ± 1.89 ^cde^	4.28 ± 0.94 ^a^	1.61 ± 0.13 ^b^	1.40 ± 0.41 ^bc^
S25095	13.85 ± 2.40 ^a^	4.25 ± 1.01 ^a^	3.35 ± 0.54 ^a^	2.10 ± 0.51 ^ab^
S25099	6.10 ± 1.76 ^de^	4.30 ± 0.98 ^a^	1.50 ± 0.56 ^b^	1.20 ± 0.39 ^c^

Note: Values are mean ± standard deviation. Different letters in the same column indicate significant differences among treatments at *p* < 0.05.

**Table 9:** Root architecture parameters of tomato under different treatments.

Treatment	Total Root Length (cm)	Root Tip Number	Mean Diameter (mm)	Surface Area (cm^2^)	Fractal Dimension
T1	634.37 ± 111.40 a	316.33 ± 42.52 a	1.86 ± 0.07 a	65.60 ± 12.51 a	1.72 ± 0.03 a
T2	407.90 ± 72.21 b	232.33 ± 39.80 b	1.56 ± 0.20 a	36.03 ± 2.98 cd	1.73 ± 0.02 a
T3	347.03 ± 53.00 b	157.33 ± 17.62 c	1.50 ± 0.13 a	29.04 ± 3.45 d	1.64 ± 0.01 b
T4	480.42 ± 61.71 b	215.00 ± 13.00 bc	1.83 ± 0.14 a	49.23 ± 9.74 b	1.68 ± 0.05 ab
T5	436.60 ± 79.49 b	220.67 ± 52.16 bc	1.77 ± 0.36 a	42.40 ± 3.30 bc	1.68 ± 0.03 ab
CK	376.77 ± 5.64 b	171.00 ± 13.89 bc	1.59 ± 0.12 a	34.46 ± 2.41 cd	1.69 ± 0.05 ab

Note: Values are mean ± standard deviation. Different letters in the same column indicate significant differences among treatments at *p* < 0.05.

## Data Availability

The original contributions presented in this study are included in the article/Supplementary Material. Further inquiries can be directed to the corresponding author.

## Supplementary Materials

The following supporting information can be downloaded at: https://www.mdpi.com/article/10.3390/plants15101486/s1, Figure S1: Indicator bacterial species and their enrichment characteristics; Figure S2: Indicator fungal species and their enrichment characteristics.

## Abbreviations

AN	Available nitrogen
AP	Available phosphorus
AK	Available potassium
CFU	Colony-forming unit
IAA	Indole-3-acetic acid
PKO	Pikovskaya medium
PGPR	Plant growth-promoting rhizobacteria
SOM	Soil organic matter
SPAD	Relative chlorophyll content
TN	Total nitrogen
