# Supplementary material for: Biomass Seedling Trays Drive Rhizosphere Microbiome Restructuring and PGPR Enrichment in Tomato
Source: Plants (Basel). 2026 May 13;15(10):1486. doi: 10.3390/plants15101486 (PMC13210904; doi:10.3390/plants15101486)
Supplement: Supplementary file 1 [file plants-15-01486-s001.zip › plants-4281707-supplementary.pdf]

Supplementary Information

Biomass Seedling Trays Drive Rhizosphere Microbiome Restructuring and PGPR Enrichment in Tomato

Jiayun Zhang, Xiangyu Zhang and Qiang Chen *

College of Resources, Sichuan Agricultural University, Chengdu 611130, China;
zhangjiayun2026@126.com (J.Z.); ZXY18180765055@126.com (X.Z.)
* Correspondence: cqiang@sicau.edu.cn

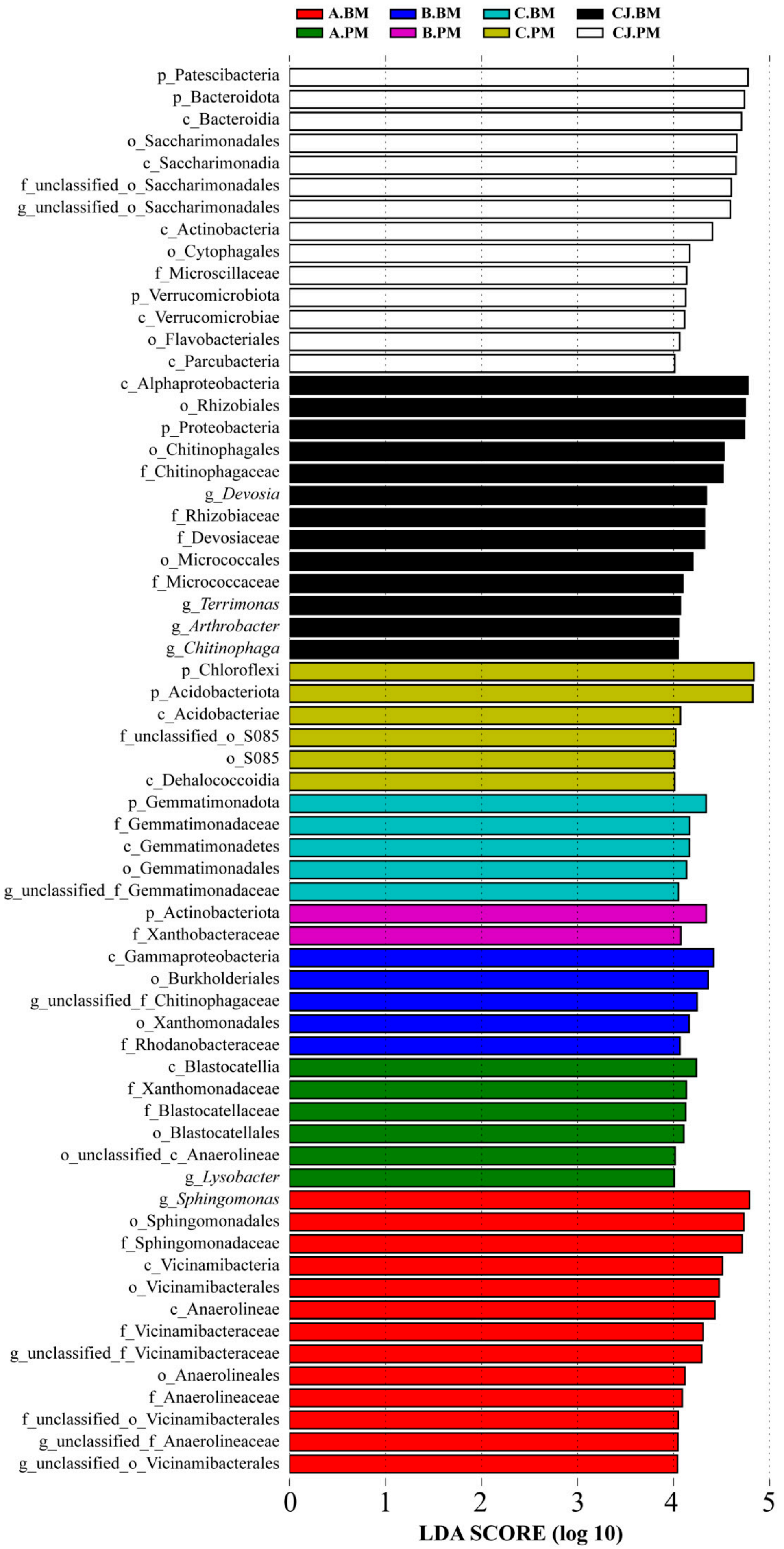

Figure S1. Indicator bacterial species and their enrichment characteristics.

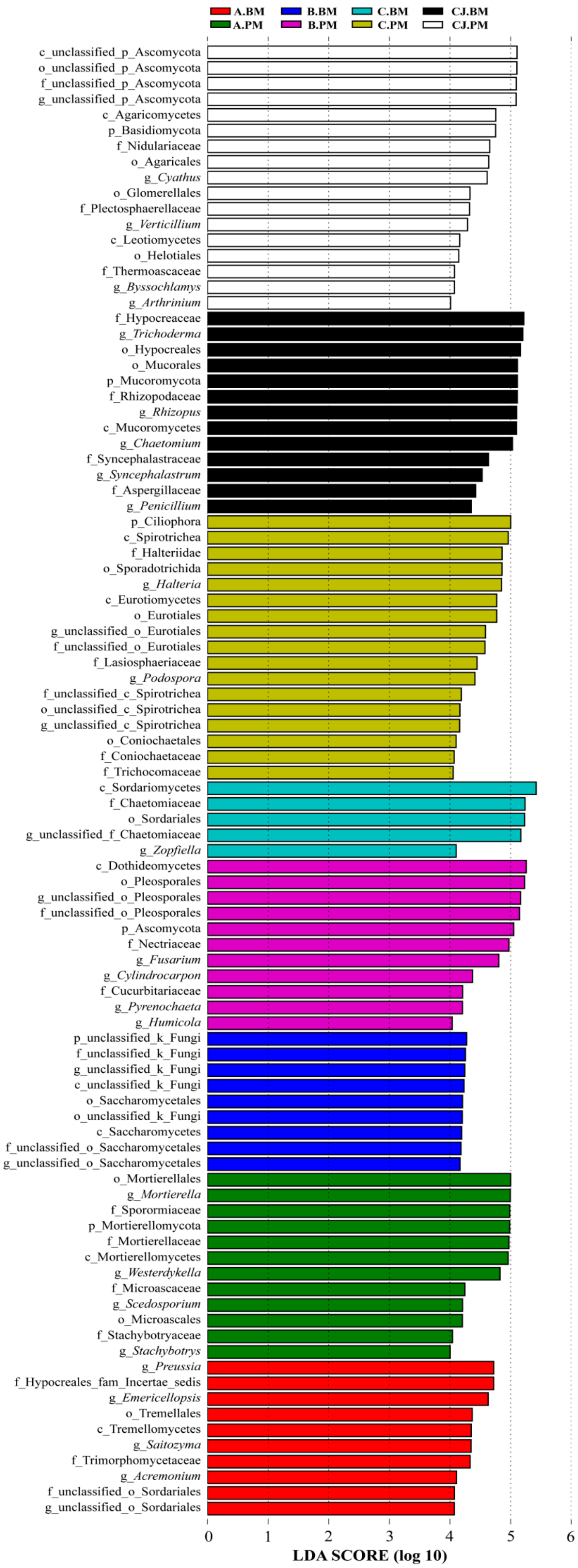

Figure S2. Indicator fungal species and their enrichment characteristics.
